# Heterogeneity of Stemlike Circulating Tumor Cells in Invasive Breast Cancer

**DOI:** 10.3390/ijms21082780

**Published:** 2020-04-16

**Authors:** Olga E. Savelieva, Liubov A. Tashireva, Evgeniya V. Kaigorodova, Angelina V. Buzenkova, Rustam Kh. Mukhamedzhanov, Evgeniya S. Grigoryeva, Marina V. Zavyalova, Natalia A. Tarabanovskaya, Nadezhda V. Cherdyntseva, Vladimir M. Perelmuter

**Affiliations:** 1 Cancer Research Institute, Tomsk National Research Medical Center, 634050 Tomsk, Russia; lkleptsova@mail.ru (L.A.T.); zlobinae@mail.ru (E.V.K.); buzenkova_av@mail.ru (A.V.B.); grigoryeva.es@gmail.com (E.S.G.); zavyalovamv@mail.ru (M.V.Z.); tarabanovskaya@inbox.ru (N.A.T.); nvch@tnimc.ru (N.V.C.); pvm@ngs.ru (V.M.P.); 2 Siberian State Medical University, 634050 Tomsk, Russia; dx.rusya@gmail.com

**Keywords:** circulating tumor cells, stemness, EMT, heterogeneity, breast cancer

## Abstract

The presence of stem and epithelial–mesenchymal-transition (EMT) features in circulating tumor cells (CTCs) determines their invasiveness, adaptability to the microenvironment, and resistance to proapoptotic signals and chemotherapy. It also allows them to fulfil the role of metastatic “seeds”. We evaluated the heterogeneity of stem CTCs by their CD44, ALDH1, and CD133 expression depending on N-cadherin expression in breast-cancer patients. A total of 38 female patients were selected for this study. CTC phenotypes were determined by flow cytometry before any type of treatment. Multiplex immunofluorescence was used for the evaluation of tumor-cell heterogeneity in primary lesions. In patients who had CD44-CD24- CTCs, a subset of cells with the expression of other stem-cell markers (CD133 and ALDH1) were detected. Expression of CD133 and/or ALDH1 may be associated with expression of N-cadherin: all populations of N-cadherin+ CTCs demonstrate stem features; in the absence of N-cadherin expression, true nonstem (CD44-CD24-CD133-ALDH1-) cells are found. The heterogeneity of stem marker expression in CTCs was observed regardless of N-cadherin expression. In our study, stromal cell-derived factor-1 (SDF-1) receptor expression in CTCs did not depend on stemlike traits, but was instead associated with N-cadherin expression. Subpopulations of tumor cells, detected both in tumors and blood, were identified. Breast cancer was characterized by pronounced interpersonal and intrapersonal heterogeneity of CTCs by the presence and combination of various stem features and N-cadherin expression. To complete the characterization of stemlike features of CTCs, we suggest the simultaneous use of the three stem markers.

## 1. Introduction

Circulating tumor cells (CTCs) are essential and informative objects for understanding the metastasis of carcinomas [1]. They come in metastasis lesions and become, for an indefinite time, disseminated tumor cells (DTCs). The fate of DTCs is twofold. In the first case, DTCs produce metastasis if they possess all characteristics of “seed” cells, and if a functional premetastatic niche exists [2,3]. In the other case, these cells become dormant if the triggers are absent or the microenvironment (niche) is deficient [4].

Disseminated dormant cells are often unavailable for research. Thus, it is necessary to pay attention to the study of biological characteristics of CTCs as archetypes of seed or dormant cells. Currently, numerous data confirmed the significant heterogeneity of the CTCs and cells in primary tumors [5]. 

Among the many heterogeneity characteristics of tumor cells, the most important are the features of epithelial–mesenchymal-transition (EMT) presence and its depth, and the stemlike properties of the cells. Those particular conditions are responsible for the most significant features of the cells, determining their resistance to apoptosis, chemoresistance, invasiveness, and adaptability to the microenvironment; eventually, they allow these cells to be metastatic seeds [6]. Research on EMT and the stem features of CTCs is difficult for many reasons. First, CTCs are very rare events. Second, a universal marker for EMT and stem states is absent. Thus, for EMT and stemness characterization, the determination of marker combinations is required. The EMT process is characterized by many features: from the epithelial state through many intermediate epithelial–mesenchymal states, to exhibiting the mesenchymal phenotype [7,8]. This process is referred to as epithelial–mesenchymal plasticity [9]. N-cadherin serves as an indicator of transitional (hybrid/metastable) EMT states [10]. Therefore, the determination of only one EMT marker can be the reason for obtaining false-negative results. 

The same situation happens with the determination of stem features. As a rule, in most studies, only one of three stem markers (CD44 and CD24, or ALDH1, or CD133) is used. The detailed description of cells with the expression of different stem markers was shown in some articles, such as the review of Paula A.D.C. and Lopes C. [11]. The CD44+CD24−/low phenotype is associated with breast cancer 1 (*BRCA1*) mutational status and basal-like tumor status [12]. ALDH1 positivity correlates with a high histological grade, HER2 overexpression, and the absence of estrogen- and progesterone-receptor expression [13]. CD133 was proven to be suitable in the identification of CSCs in triple-negative breast cancers through several in vivo studies [14]. In addition, CD133 was detected in circulating tumor cells in patients with triple-negative breast cancer [15]. 

Obviously, the absence of one of these markers cannot be interpreted as the absence of stem features. There is a paucity of data considering the significance of stem-marker monoexpression, and their combination in a certain stem tumor cell for distant-metastasis development.

Only a small fraction of CTCs (by various data, from 0.01% to 0.1%) can be the source of metastases [16,17]. Not all EMTs or stem exhibitions are enough for seed properties. This may explain the low metastatic efficiency of CTCs. The intermediate epithelial–mesenchymal (hybrid, metastable) state is more efficient to metastasis, but not the extreme variants of EMT with epithelial or mesenchymal phenotypes [18,19,20]. 

Answers can be obtained in the simultaneous determination of multiple CTC parameters. The tool for this procedure can be sequencing. Despite the informative value and importance of the method, the assessment of the characteristics of only one cell remains difficult. Additionally, gene-expression identification (e.g., EpCAM) does not provide information regarding the functional significance that is associated with its cell localization (membrane, cytoplasm, and nucleus). Detection of gene transcripts associated with EMT without corresponding protein identification cannot provide exact data regarding the EMT phase (especially the hybrid/intermediate state). Flow-cytometry methods are more suitable for the multiparametric assessment of CTCs compared with sequencing. The technology of multiplex fluorescent microscopy has similar capacities.

We focused on the assessment of CTC heterogeneity in invasive-breast-cancer patients and the study of its stemlike features by the simultaneous usage of three markers (CD44 and CD24, ALDH1, or CD133), depending on N-cadherin expression.

## 2. Results

### 2.1. Phenotypic CTC Heterogeneity 

**Figure 1:**
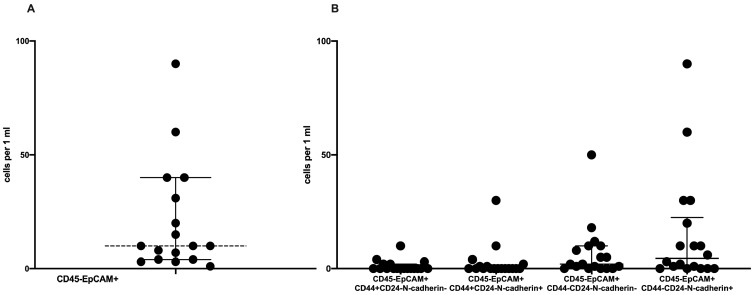
Counts of circulating tumor cells (CTCs) in breast-cancer patients. (**A**) all EpCAM-positive CTCs; (**B**) CTC subsets by stem and epithelial–mesenchymal-transition (EMT) features.

Circulating tumor cells were detected in 47.36% (18 out of 38) of the cases. The average number of CTCs was equal to 10 (4–40) in 1 mL of peripheral blood (Figure 1A).

We assessed the frequency of CTCs depending on the combination of stem and EMT markers (Figure 1B). CTCs without EMT and stem features (CD45-EpCAM+CD44-CD24-N-cadherin-) were detected in 72.2% (13/18) of the cases. Nonstemlike CTCs with EMT characteristics (CD45-EpCAM+CD44-CD24-N-cadherin+) were present in patients’ blood with the same rate. Stemlike CTCs with and without EMT features (CD45-EpCAM+CD44+CD24-N-cadherin+ and CD45-EpCAM+CD44+CD24-N-cadherin-, respectively) were detected in 33.3% (6/18) and in 27.7% (5/18) of the cases, respectively. Nonstemlike CTCs without and with EMT features were present in most cases and numerous among other CTCs subsets (2.0 (0.0–10.0) and 4.5 (0.0–22.5) cells in 1 mL of peripheral blood; Figure 1B). The number of stemlike CTCs was lower (Figure 1B).

**Figure 2:**
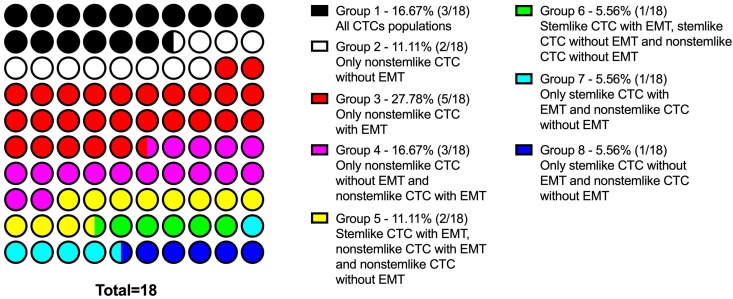
Contingency of personal CTCs profile in breast-cancer patients. Interpersonal heterogeneity characterized with various combinations of CTCs with and without stemlike and/or EMT features. Eight patient groups with a combination of various CTC phenotypes presented.

Interpersonal heterogeneity was characterized with various combinations of CTCs with stemlike and EMT features or their absence (Figure 2). Three patients all had studied CTC subsets (Group 1). Two patients only had CTCs without stem and EMT features (Group 2). Five patients only had nonstemlike CTCs with EMT features (Group 3). Three patients had nonstemlike CTCs with and without EMT features (Group 4). Two patients had three CTC subsets: stemlike CTCs with EMT, nonstemlike CTCs with EMT, and stemlike CTCs without EMT (Group 5). Other combinations of the CTC subsets were found in three patients (Groups 6–8; Figure 2).

### 2.2. Frequency of Tumor Cells with Combinations of CD44 and N-Cadherin Expression in Primary Tumors and Peripheral Blood

**Figure 3:**
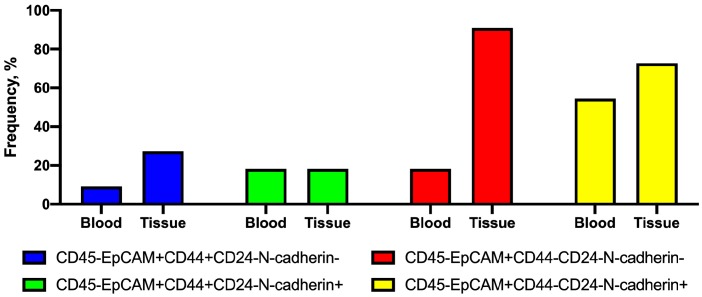
Frequency of tumor cells with different CD44 and N-cadherin expression in primary tumors and peripheral blood.

First, the frequency of tumor-cell subsets in blood and primary tumors was similar. The CD45-EpCAM+CD44-CD24-N-cadherin- cell subtype was the exception; its frequency in tissue was higher than in blood (Figure 3).

**Figure 4:**
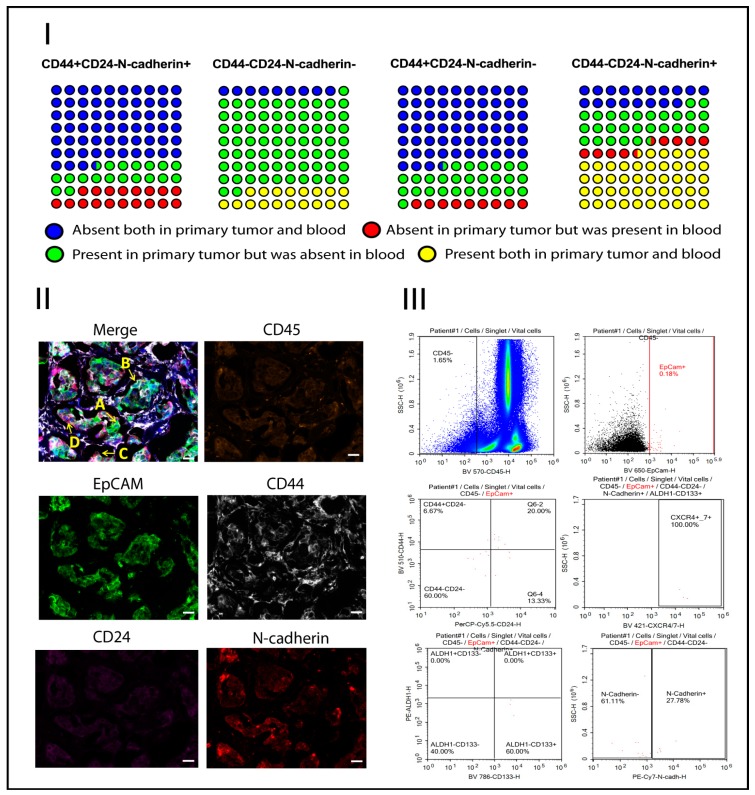
Matching tumor-cell phenotypes of primary tumor and peripheral blood: (I) variants of tumor-cell presence in primary tumor and peripheral blood; (II) subsets of tumor cells with and without EMT and/or stem features in primary breast cancer: A—CD45-EpCAM+CD44-CD24-N-cadherin-, B—CD45-EpCAM+CD44+CD24-N-cadherin-, C—CD45-EpCAM+CD44+CD24-N-cadherin+, D—CD45-EpCAM+CD44-CD24-N-cadherin+ (scale bar, 40 um); (III) subsets of CTCs with and without EMT and/or stem features in peripheral blood.

The presence of four cell subsets in the primary tumors and peripheral blood of the same patients was also assessed. Four possible variants were considered: (1) the subset was absent both in the primary tumor and the blood; (2) the subset was present in the primary tumor but absent in the blood; (3) the subset was absent in the primary tumor but present in the blood; and (4) the subset was present both in the primary tumor and in the blood (Figure 4).

First, the difference between the four variants that were described above, in the presence/absence of CD44+CD24- cell subsets in the primary tumor and peripheral blood, was not significant in groups with and without N-cadherin expression (*p* = 0.424). In addition, in 63.4% of cases, these subsets were not detected either in the primary tumor or in the peripheral blood. These subsets of CTCs were not simultaneously observed in the primary tumor and the blood. In one case, CD44+CD24- cell subsets were detected only in the primary tumor, and in other cases, only in the blood.

Different results were obtained in comparison of the presence of nonstemlike (CD44-CD24-) tumor cells in the primary tumor and peripheral blood. The difference was at the trend (*p* = 0.086) depending on N-cadherin expression. Compared with stemlike CTC subtypes, when these cells were absent both in the primary tumor and peripheral blood, the number of those cases was lower. The frequency of cases with the presence of N-cadherin-negative CD44-CD24- cells in the tumor and their absence in the blood was higher (in 72.7% of the cases) compared with N-cadherin-positive cells. These cells were detected simultaneously in the primary tumor and peripheral blood in 18.18% of cases compared with CD44+CD24-N-cadherin- CTCs (Figure 4). 

In contrast to these results, the CD44-CD24-N-cadherin+ CTC subset was detected more rarely in the primary tumor (in 27.7% of the cases), and was absent in the blood. These cells were detected both in the tumor and peripheral blood with a high rate (43.45% of the cases).

### 2.3. Combinations of Stem Markers in CTC Subsets

**Figure 5:**
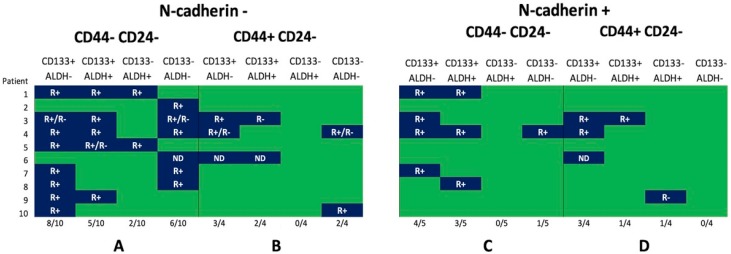
CTC profiles depended on N-cadherin expression and combination of stem markers in breast-cancer patients. Frequency of (**A**) nonstemlike CTCs without EMT features; (**B**) stemlike CTCs without EMT features; (**C**) nonstemlike CTCs with EMT features; and (**D**) stemlike CTCs with EMT features. R, CXCR4 and/or CXCR7 expression; ND, no data.

The combinations of stem markers (CD44, CD133, and ALDH1) in N-cadherin-positive and N-cadherin-negative CTCs were assessed in 10 out of 18 patients. Sixteen combinations of CTC phenotypes were analyzed in total (Figure 5). CTCs with various stem properties were detected in 90% of the cases. In addition, significant intrapersonal heterogeneity of CTCs in stemness characteristics was observed.

The primary goal of this study was the comparison of stem-marker (ALDH1 and CD133) expression in CD44-CD24- and CD44+CD24- CTC subsets depending on the presence of EMT features (N-cadherin expression).

The number of various CTC phenotypes in one patient varied from 1 out of 16 to 9 out of 16. The CD45-EpCAM+N-cadherin-CD44+CD24-CD133-ALDH1+, CD45-EpCAM+N-cadherin+CD44+CD24-CD133-ALDH1-, and CD45-EpCAM+N-cadherin+CD44-CD24-CD133-ALDH1+ CTC subsets were not detected in any cases (Figure 5).

Cells not expressing other stem markers, such as ALDH1 and CD133, were detected in 20% of the cases among the 10 patients with the CD45-EpCAM+N-cadherin-CD44-CD24- CTCs. In other patients among the CD45-EpCAM+N-cadherin-CD44-CD24- cells. Stem cells with only CD133 expression (80% of the cases), only ALDH1 expression (20% of the cases), and CD133 and ALDH1 coexpression (50% of the cases) were present in the peripheral blood (Figure 5A). 

Among five patients with CD45-EpCAM+N-cadherin+CD44-CD24- CTCs, the other stem markers were present in all cases. CD133 monoexpression was observed in 80% of the cases. CD133 and ALDH1 coexpression in CTCs was detected in 60% of the cases. Cells with ALDH1 monoexpression were not detected (Figure 5C).

Among the four patients with the CD45-EpCAM+N-cadherin-CD44+CD24- CTCs, cells with CD44 monoexpression were observed in 25% of the cases. CD44 and CD133 coexpression was present in 75% of the cases, while CD44 and ALDH1 coexpression was absent. Triple-positive stem cells (CD44+CD133+ALDH1+) were detected in half of the cases (Figure 5B).

The CD45-EpCAM+N-cadherin+CD44+CD24- CTC subset, in all cases, expressed other stem markers. CD44 and CD133 coexpression was present in 75% of the cases, and CD44 and ALDH1 coexpression was present in 25% of the cases. Triple-positive CTCs were observed in 25% of the cases (Figure 5D).

SDF-1 receptors (C-X-C chemokine receptor types 4 and 7; CXCR4 and CXCR7) were observed in the majority of assessed CTC subsets. Their expression was present both in CTCs with stem features and/or EMT, and in cells without these characteristics. Most N-cadherin-positive CTCs expressed CXCR4 and CXCR7, while, in N-cadherin-negative CTCs, cells without expression of these receptors were more frequent among several subsets of stemlike and nonstemlike cells. 

### 2.4. Association of CTC Subset Frequencies with Clinicopathological Parameters

The association of CTCs with clinicopathological parameters of invasive breast carcinoma of no special type (NST) was assessed. The comparison of clinicopathological parameters in groups of patients without CTCs and with various CTC subtypes was carried out. Significant difference in clinicopathological parameters in groups of CTC-positive and -negative patients was not observed (Table 1). 

**Figure 6:**
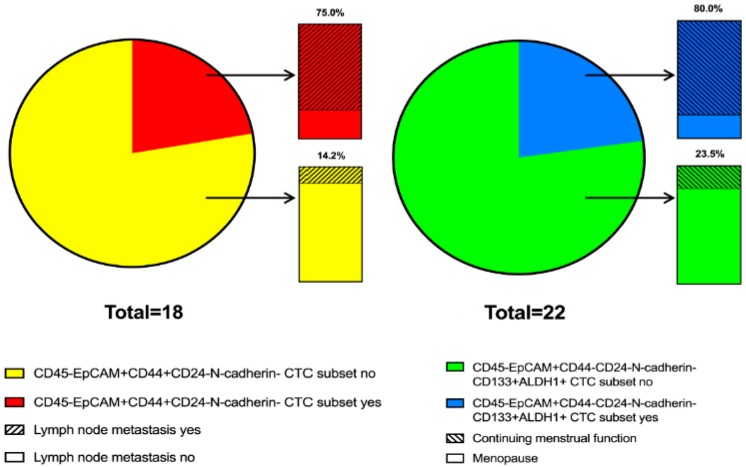
Association of CTC subsets with lymph-node metastasis and menstrual function in breast-cancer patients.

The association of various CTC subsets with age, tumor size, menstrual function, grade, molecular type, and stage of the disease is not shown. At the same time, the rate of lymph-node metastases in patients with the CD45-EpCAM+CD44+CD24-N-cadherin- CTC subset was equal to 75% (3/4); in the absence of such a CTC subset, lymph-node metastases were developed in 14.3% (2/14) of patients (*p* = 0.044). The CD45-EpCAM+CD44-CD24-N-cadherin-CD133+ALDH1+ CTC subset was more often present in patients with continuing menstrual function (80% (4/5) compared to 23.5% (4/17), *p* = 0.039; Figure 6). 

## 3. Discussion

Assessment of stemness and EMT characteristics allows us to reveal the significant intrapersonal heterogeneity of CTCs. We observed various combinations of CTC phenotypes in each patient. Cases with the presence of only cells with stemlike features among all CTCs were not detected. The intravasation ability of primary tumor cells is the key process in the mechanism of CTC generation. Not all tumor cells have intravasation capability. This capability was shown in different model systems [21]. 

Tumor cells must possess the capacity to form the tumor microenvironment of metastasis (TMEM) with a perivascular macrophage and an endothelial cell [22], or express EGF receptors for hematogenous dissemination [23]. The presence of EMT also plays an important role in intravasation [10,24].

It is likely that there are several variants whereby we can explain the fact that we detected a wide range of CTC phenotypes (without stemness and EMT characteristics, with stemness characteristics by one or several markers, with the expression of EMT marker N-cadherin, or a combination of stemness and EMT features). It is conceivable that the intravasation process is accessible for cells with various initial phenotypes. This suggestion was confirmed by data regarding the presence of intravasation mechanisms of tumor cells deprived of invasive characteristics [21,25].

The other possible explanation for CTC heterogeneity lies in the fact that only a small number of tumor-cell subsets have the ability for intravasation. CTC phenotype change occurs in circulation, which leads to large degree of heterogeneity. However, this variant of CTC heterogeneity explanation is also doubtful. First, CTCs are present in the blood for a short period (1–3 h) [26]. Secondly, they are not attached to the cell matrix, and this may restrict their function. The third variant is based on how only several tumor-cell phenotypes with intravasation ability can undergo phenotype changes during migration in the perivascular matrix and through the vessel wall. These changes may depend on the initial cell phenotype.

Clarification of the mechanism of CTC heterogeneity origin is very important. It allows us to understand whether CTC generation is an independent factor increasing heterogeneity and potential tumor aggressiveness. This clarification would also explain the imbalance of primary-tumor-cell subsets and CTCs. Apparently, a mismatch of subset composition of primary tumor cells and CTCs is more probable than their sameness. This may be explained by variability in intravasation ability, survival in peripheral blood, and extravasation capacity. That is why several tumor-cell subtypes may be present in a tumor but absent among CTCs.

Matching cell phenotypes in the primary tumor and the blood allowed us to assume that stemlike CD44+ tumor cells have decreased intravasation capacity and CTC generation regardless of N-cadherin expression. In contrast, nonstemlike CD44- tumor cells have a significant intravasation ability that requires a certain epithelial–mesenchymal status (expression of N-cadherin). Moreover, the absence of this EMT marker is associated with high-frequency (45.5%) detection of these cells among CTCs. Consequently, the intravasation of nonstemlike tumor cells is associated with the combination of EMT features.

The CTC count is the prognostic marker of distant metastases [16,17]. In this regard, CD45-EpCAM+CD44-CD24-N-cadherin+ cells are the most likely candidates for seed cells due to their high frequency in primary tumors and peripheral blood (Figure 4). We demonstrated by the other stem markers that this CTC subtype is heterogeneous. Therefore, we had to determine which phenotypic variant of EpCAM+CD44-CD24-N-cadherin+ cells was more likely to possess seed-cell properties.

Main data concerning the functional differences of stem tumor cells are received in most cases using only one of the stem markers. Data concerning the functional properties of stemlike cells with the expression of two or more markers are less common. During the assessment of CTCs, these data were even more insufficient. The detection of various kinds of stem-marker coexpression is necessary, as data regarding the functional differences of tumor cells expressing various stem markers are now present. 

Thus, CD44 is associated with breast-cancer progression: cell adhesion, behavior, motility, morphology, and tumorigenesis [27]. CD44 also regulates Th1 survival, memory function, IL-17, and IFN-α production by T cells [28]. Although CD44+CD24−/low stem cells are scrutinized, the results of their clinical significance are indeterminate. Thus, there was no significant correlation of CD44+CD24−/low stem cells and tumor progression, as well as recurrence, disease-free survival (DFS), or overall survival (OS) [29]. CD44+/CD24−/low phenotype was associated with *BRCA1+* and basal-like tumor status. However, despite its association with increased poor prognostic features, it was not able to predict OS [12].

ALDH1 expression correlates with clinical outcome in many cancers. High levels of ALDH1 correlate with a poor outcome in ovarian, colorectal, prostate, rectal, and lung cancers, and glioblastoma [30]. The functional significance of CD44 and ALDH1 is not equivalent. A high CD44/CD24 ratio was more related to cell proliferation and tumorigenesis, while ALDH1+ was a stronger indicator for metastasis [31].

In our study, cells with ALDH1 monoexpression were absent. This was not a coincidence, as ALDH1 expression is associated with the epithelial type of tumor cells that do not usually express N-cadherin [32]. 

Data regarding the association of various stem markers with epithelial and mesenchymal phenotypes are interesting. Aktas B. et al. discussed the association between various features of EMT and ALDH1 expression [33]. May C.D. et al. assumed that the combination of ALDH1 and CD44+CD24-/low could be used for detecting EMT-associated tumor stem cells of breast cancer with high tumorigenic capacities [34]. CD44+/ALDH1+ cells were probably predominantly in a quiescent state (negativity for Ki-67 proliferation marker) [35]. In several studies, in breast cancer, CD44+CD24- mesenchymal CSCs were found at the invasive front of the tumor, while epithelial-like ALDH1+ CSCs were mostly detected in the inner regions [36]. 

Initially, ALDH1+ CSCs were considered as epithelial-like cells, but these cells were later shown to be closer to the E/M-phenotype hybrid and have several genes with a triple-negative breast-cancer signature [37]. Recent studies identified distinct epithelial-like (E) ALDH1+ CSCs, mesenchymal-like (M) CD44+CD24− CSCs, and hybrid E/M ALDH1+CD44+CD24− CSCs [33]. CD133 is a stemness marker that is related to increased tumor-initiating ability, tumor progression, metastasis, therapeutic resistance, and cancer recurrence in numerous types of cancer [38]. Apparently, CD133 expression is not associated with invasiveness, as it is a malignancy indicator for both invasive and noninvasive breast cancers [39].

Thus, the detection of various stem-marker coexpressions is important for a personal assessment of metastasis risk, therapy resistance, and other processes associated with stem cells.

The assessment of various stem-marker coexpressions allows us to conclude that, among CD44- CTCs, cells with other stem markers can be present. Thus, only in 60% of cases were CD44-negative cells nonstem. Different stemness variants were observed in 40% of cases. Consequently, it is not possible to make suggestions regarding the absence of stem features in CTCs using only one stem marker. 

During the study of stem characteristics in tumor cells, it is necessary to use at least these three markers (CD44/CD24, CD133, and ALDH1). On the other hand, due to tumor-stem-cell heterogeneity, it is necessary to search for a universal stem marker. This marker must be indispensably associated with the main characteristics of stem cells—the ability of asymmetrical division and a long-lasting period of the quiescent state. CD44-positive cells also had other stem markers in 80% of cases. Only in 20% of cases was CD44 expression the only stem feature.

Cells with ALDH1 and CD44 coexpression, and triple-positive cells appeared more rarely than cells with CD133 and CD44 coexpression. The same pattern of ALDH1 and CD133 expression was observed in the CD44-negative cell subset. Cases with ALDH1 expression appeared more rarely. The frequency of cells with different stem features among N-cadherin-negative and -positive subsets did not significantly differ. The high frequency of CXCR4 and CXCR7 expression in various N-cadherin-positive CTC subsets also attracted attention. As far as SDF-1 is an important chemoattractant for seed-cell adhesion in the premetastatic niche and is also a factor conducive to the appearance of stem characteristics in tumor cells [40], coexpression of its receptors with N-cadherin is, apparently, expected.

There are incontestable facts regarding the identity of certain molecular features detected in cells in EMT and stem processes [9,33]. Moreover, the EMT process itself can lead to the acquisition of stem characteristics by cells. In this context, a situation is expected where it is difficult to separate EMT and stem characteristics in tumor cells. Our results indicated that stem and EMT features may exist independently of each other. N-cadherin expression, which is attributed to hybrid (intermediate/metastable) EMT variants, can be observed both in stemlike CTCs and in CTCs without any of the three used stem-cell markers.

### Summary

- CTCs were detected in 47% of patients with invasive carcinoma of no special type (NST).
- CTC population in NST patients is characterized by significant interpersonal heterogeneity: there are cells with only stem features, ones with only EMT features, with a combination of these properties, and with the absence of both features.
- Interpersonal heterogeneity of CTCs in patients with NST is characterized by different frequency of occurrence of each subsets and different combinations of subsets in each patient.
- Depending on the presence of different subsets of tumor cells in the primary tumor and among CTCs, the following patterns were revealed:
  ○ stem cells, regardless of the expression of N-cadherin, were detected either only in the tumor or only among CTCs, and there were no cases of the simultaneous detection of such cells in the tumor and in the peripheral blood;
  ○ nonstem cancer cells were simultaneously present in the tumor and in the peripheral blood, and more often, this combination was found in the presence of EMT features (positive N-cadherin expression).
- In patients who had CD44-CD24- CTCs, the subset of cells with the expression of other stem-cell markers (CD133 and ALDH1) was detected.
- The expression of CD133 or/and ALDH1 may be associated with the expression of N-cadherin:
  ○ all populations of N-cadherin+ CTCs demonstrated stem features;
  ○ in the absence of N-cadherin expression, true nonstem (CD44-CD24-CD133-ALDH1-) cells were found.
- The presence of CD45-EpCAM+CD44+CD24-N-cadherin- cells in NST patients is associated with lymph-node metastasis.

## 4. Materials and Methods 

### 4.1. Samples

The prospective study included 38 patients with NST T2-4N0-3M0, admitted for treatment to Cancer Research Institute, Tomsk National Research Medical Center. No-one was treated by neoadjuvant chemotherapy. Venous ethylenediaminetetraacetic acid (EDTA) blood samples were taken one to two days before surgical intervention. The study was approved by the Local Committee for Medical Ethics of our institute (17 June 2016, the approval No. 8), and informed consent was obtained from all patients prior to analysis. The clinicopathological parameters of the patients with breast cancer are presented in Table 1.

### 4.2. CTC Enrichment and Flow Cytometry

As CTCs are extremely rare events and count only several cells per 1 mL of blood, an enrichment of the sample is an appropriate step in sample preparation. We analyzed available CTC-enrichment methods, such as the CellSearch system and microfluidics-based approaches. The CellSearch system is based on the isolation of CTCs with the surface expression of EpCAM; however, according to modern concepts, CTCs are a heterogeneous population and are represented by cells with different phenotypes. Moreover, some CTCs are characterized only by the intracellular expression of EpCAM. Accordingly, the use of this approach would lead to a significant loss of CTCs. 

Microfluidics methods are based on the isolation of CTCs by size, as CTCs can significantly change their morphology and have smaller sizes in the EMT process. In this way, CTCs with such characteristics were also not included in the enriched sample. As EDTA accelerates the deposition of red blood cells, it is possible to obtain plasma containing many nonerythrocyte cells [41]. Therefore, to avoid the loss of target cells, we chose this method for producing a cell concentrate by incubating the EDTA blood in a thermostat at 37 °C for 90 min and analyzing the total number of nucleated cells obtained from 12 mL of whole blood. The obtained cell concentrate was centrifuged, and the cell pellet was resuspended in 1 mL of staining buffer (Sony Biotechnology, Tokyo, Japan).

We evaluated the phenotypic characteristics of CTCs through flow cytometry (Novocyte 3000, ACEA Biosciences, San Diego, CA, USA) using a monoclonal-antibody cocktail. Cells were stained in two steps: surface markers were first stained, and then intracellular markers were stained. To avoid the unspecific binding of antibodies, we utilized Human TruStain FcX™ Fc Receptor Blocking Solution (Biolegend, San Diego, CA, USA). After blocking, monoclonal antibodies were added: 5 μL of BV570-anti-CD45 (clone HI30, mouse IgG1, Sony Biotechnology, San Jose, CA, USA), BV650-anti-EpCAM (clone 9C4, mouse IgG2b, Sony Biotechnology, San Jose, CA, USA), PE-Cy7-anti-N-cadherin (clone 8C11, mouse IgG1, Sony Biotechnology, San Jose, CA, USA), BV510-anti-CD44 (clone G44-26, mouse IgG2b, BD Horizon, Franklin Lakes, NJ, USA), PerCP-Cy5.5-anti-CD24 (clone ML5, mouse IgG2a, Sony Biotechnology, San Jose, CA, USA), BV786-anti-CD133 (clone 293C3, Mouse BALB/c IgG2b, κ, BD Pharmingen, San Jose, CA, USA), BV421-anti-CXCR4 (clone 12G5, Mouse IgG2a, Sony Biotechnology, San Jose, CA, USA) and BV421-anti-CXCR7 (clone 10D1, Mouse C57BL/6 IgG2a, κ, BD Horizon, Franklin Lakes, NJ, USA). 

Viable cells were identified by the use of 7-amino-actinomycin D (7-AAD, Sony Biotechnology, San Jose, CA, USA). The corresponding isotype control at the same concentration was added to the control sample. After incubation, cells were lysed using an OptiLyse buffer (Beckman Coulter, Marseille, France) and washed in Cell Wash buffer (BD Biosciences, San Jose, CA, USA). For intracellular stains, cells were permeabilized using BD Cytofix/Cytoperm (BD Biosciences, San Jose, CA, USA). After permeabilization and washing, monoclonal antibodies were added: BV650-anti-EpCAM (clone 9C4, mouse IgG2b, Sony Biotechnology, San Jose, CA, USA) and PE-anti-ALDH1A1 (clone 03, Mouse IgG1, Sino Biological, Wayne, PA, USA).

The gating of the cell populations was carried out on the basis of determining the parameters of forward scatter light signals (FSC) and side scatter light signals (SSC). Cells were then analyzed for fluorescence in density- and dot-plot modes. Positive cells were counted per 1 mL of whole blood.

### 4.3. CTC Spiking Experiment

Blood specimens (12 mL with EDTA) for three spiking experiments were drawn under informed consent from healthy donors and breast-cancer patients at the Cancer Research Institute, Tomsk National Research Medical Center, according to a protocol approved by the Local Committee for Medical Ethics. The whole blood (100 μL) of a healthy donor was spiked with a known number (range, 18–33) of CTCs, which were isolated using a cell sorter, MoFlo XDP, with Summit software (Beckman Coulter, Miami, FL, USA). For this procedure, stabilized EDTA venous blood was incubated with fluorochrome-labeled monoclonal antibodies to CD45 (PE/Cy7, mouse IgG1, clone HI30, BD Pharmingen, San Jose, CA, USA), CD44 (APC-H7, mouse IgG2b, clone G44-26, BD Pharmingen, San Jose, CA, USA), CD24 (PerCP-Cy5.5, mouse IgG2a, clone ML5, BD Pharmingen, San Jose, CA, USA), EpCAM (FITC, mouse IgG1, clone EBA-1, BD Pharmingen, San Jose, CA, USA), and CD325 (N-cadherin) (PE, mouse IgG1, clone 8C11, BD Pharmingen, San Jose, CA, USA). Isotype control antibodies were used for a negative control. Three samples of the enriched blood were counted on a flow cytometer. The results of the flow-cytometry assay ranged from 89% to 103% (CV 6%).

### 4.4. TSA-Assistance Multiplex Immunofluorescence

First, using a uniplex Multiplex immunofluorescence (IF) assay, we optimized all antibodies and generated spectral libraries for multiplex IF image analysis. Uniplex IF staining was performed by using the BondRXm staining system (Leica Microsystems, GmbH, Germany) and individual TSA-conjugated fluorophores from the Opal 7 kit (catalogue #NEL797001KT; PerkinElmer, Waltham, MA, USA). The following dilutions of antibodies were used: Anti-EpCAM (1:1000, clone E144, Abcam, UK), Anti-CD24 (1:100, clone SN3b, Thermo, Waltham, MA, USA), Anti-CD44 (1:100, clone 156-3C11, Thermo, Waltham, MA, USA), Anti-N-cadherin (1:500, clone EPR1791-4, Abcam, Cambridge, UK), and Anti-CD45 (1:10, clone 2B11+, Agilent, Santa-Clara, CA, USA). 

To detect antibody staining, the following fluorophores were used: Opal 520, Opal 540, Opal 570, Opal 620, and Opal 650 (dilution 1:100). The slides were counterstained with DAPI for 5 min and mounted with VECTASHIELD Hard Set (Vector Labs, Burlingame, CA, USA). All primary antibodies were optimized using control tissues. Multiplex IF was also performed by using the BondRXm staining system (Leica Microsystems, GmbH, Germany). Slides were scanned using the PerkinElmer Vectra (Vectra 3.0.3; PerkinElmer, Waltham, MA, USA). Tissue imaging and analysis were performed using inForm Advanced Image Analysis software (inForm 2.1.1 and 2.2.1; PerkinElmer, Waltham, MA, USA) according to the recommendations [42].

The frequency and count of the following cell populations were scored: CD45-EpCAM+CD44-CD24-N-cadherin-; CD45-EpCAM+CD44+CD24-N-cadherin-; and CD45-EpCAM+CD44-CD24-N-cadherin+; and CD45-EpCAM+CD44+CD24-N-cadherin+. 

### 4.5. Statistical Analysis 

All statistical analyses were performed using GraphPad Prism version 8.3.0 (GraphPad Software, San Diego, CA, USA). Obtained data were processed using variation statistics. The assessment of the normal distribution of the results was performed using the Shapiro–Wilk test. The significance of differences was assessed using the nonparametric Mann–Whitney test (for independent samples) and the Wilcoxon (Z) test (for dependent samples). Data are presented as the median (Me) and the upper and lower quartiles (Q1–Q3). Two-sided *p*-values of <0.05 were considered statistically significant.

## 5. Conclusions

We demonstrated prominent interpersonal and intrapersonal CTC heterogeneity. Clearly, the grade of heterogeneity depended on the panel of markers used in the CTC assessment. Thus, a similarity of the CTC pattern was found in 18 patients with the use of five CTC markers. We did not observe any similarity in the CTC patterns of 10 patients with the use of seven markers. Significant heterogeneity among CTCs with stemlike features was manifested by the different variants of CD44/CD24, CD133, and ALDH1 marker coexpression. Moreover, different variants of such stemlike features were observed in the presence and absence of N-cadherin expression. EMT characteristics were associated with the expression of SDF-1 receptors, which highlighted the association of EMT and the mechanisms providing the fixation of tumor cells in the premetastatic niche.

The observed association of N-cadherin expression with the highest frequency of simultaneous detection of CD44-CD24-N-cadherin+ cells in tumors and the blood explained the significance of the EMT process in intravasation. Stemlike cell heterogeneity clarified the necessity of the simultaneous use of several markers. 

We understand that the interpretation of our results has certain limitations due to an insufficient number of cases. Additionally, the prospective character of the study did not allow us to define the association of our results with overall and disease-free survival at this stage. 

Determining the functional characteristics of tumor cells with various combinations of stem-marker expressions and the role in metastases is the most important task in future research. It is also necessary to answer the following questions. Do cancer stem cells have the same plasticity as cells in the EMT state? Does this plasticity mean a sequential change in the expression of the stem markers, and what is its functional significance? To what extent are CTCs with various combinations of stem markers valid as seed or assistant cells promoting metastasis development?

## Figures and Tables

**Table 1:** Clinicopathological parameters of breast-cancer patients.

Clinicopathological Parameters		General Group	CTC–	CTC+
Age (year)	35–50	14/38 (37%)	6/38 (16%)	8/38 (21%)
	>50	24/38 (63%)	14/38 (37%)	10/38 (26%)
Molecular type of breast cancer	Luminal A	13/38 (34%)	9/38 (24%)	4/38 (10%)
	Luminal B	22/38 (58%)	10/38 (26%)	12/38 (31%)
	HER2-positive	1/38 (3%)	0/38 (0%)	1/38 (3%)
	Triple-negative	2/38 (5%)	1/38 (3%)	1/38 (3%)
Menstrual function	Premenopausal	13/38 (34%)	7/38 (19%)	6/38 (16%)
	Postmenopausal	25/38 (66%)	13/38 (34%)	12/38 (31%)
Tumor size	<20 mm	15/38 (39%)	7/38 (19%)	8/38 (21%)
	20–50 mm	23/38 (61%)	13/38 (34%)	10/38 (26%)
Stage	I	13/38 (34%)	6/38 (16%)	7/38 (18%)
	IIA	19/38 (50%)	11/38 (29%)	8/38 (21%)
	IIB	5/38 (13%)	2/38 (5%)	3/38 (8%)
	No data	1/38 (3%)	1/38 (3%)	0/38 (0%)
Tumor grade	1	5/38 (13%)	2/38 (5%)	3/38 (8%)
	2	25/38 (66%)	13/38 (34%)	12/38 (32%)
	3	3/38 (8%)	2/38 (5%)	1/38 (3%)
	No data	5/38 (13%)	3/38 (8%)	2/38 (5%)
Estrogen receptor	Positive	35/38 (92%)	19/38 (50%)	16/38 (42%)
	Negative	3/38 (8%)	1/38 (3%)	2/38 (5%)
Progesterone receptor	Positive	28/38 (74%)	14/38 (37%)	14/38 (37%)
	Negative	10/38 (26%)	6/38 (16%)	4/38 (10%)
HER2/neu	Positive	7/38 (18%)	3/38 (8%)	4/38 (10%)
	Negative	31/38 (82%)	17/38 (45%)	14/38 (37%)
Ki67 expression	<20%	13/38 (34%)	9/38 (24%)	4/38 (10%)
	>20%	25/38 (66%)	11/38 (29%)	14/38 (37%)
Lymph-node status	Yes	28/38 (74%)	14/38 (37%)	14/38 (37%)
	No	7/38 (18%)	3/38 (8%)	4/38 (10%)
	No surgery	3/38 (8%)	3/38 (8%)	0/38 (0%)
Distant metastasis	Yes	0/38 (0%)	0/38 (0%)	0/38 (0%)
	No	38/38 (100%)	20/38 (53%)	18/38 (47%)
Intraoperative radiotherapy	Yes	22/38 (58%)	11/38 (29%)	11/38 (29%)
	No	11/38 (29%)	5/38 (13%)	6/38 (16%)
	No data	5/38 (13%)	4/38 (10%)	1/38 (3%)

## Abbreviations

EMT	Epithelial–mesenchymal transition
CTCs	Circulating tumor cells
ALDH1	Aldehyde dehydrogenase 1
DTCs	Disseminated tumor cells
NST	Invasive breast carcinoma of no special type
TMEM	Tumor microenvironment of metastasis
